# Supplementary material for: A live attenuated-vaccine model confers cross-protective immunity against different species of the Leptospira genus
Source: eLife. 2021 Jan 26;10:e64166. doi: 10.7554/eLife.64166 (PMC7837694; doi:10.7554/eLife.64166)
Supplement: Supplementary file 1. [file elife-64166-supp1.docx]

**Supplementary Table 1.** *Leptospira* strains used in this study for challenge after vaccination with L1-130 *fcpA^-^* mutant

| **Species** | **Serovar** | **Strain** | **LD_50_^£^** | |
| --- | --- | --- | --- | --- |
|  |  |  | **Intraperitoneal** | **Conjunctival** |
| *L. interrogans* | Copenhageni | Fiocruz L1-130 | <10 | 2.15 x 10^6^ |
|  | Canicola | Kito | <10 | 1.78 x 10^7^ |
|  | Pomona | PO-06-047 | <10 | <10^8^ |
|  | Manilae | L495 | <10 | 2.15 x 10^7^ |
| *L. kirschneri* | Grippotyphosa | RM-52 | <10^3^ | 5 x 10^7^ |
| *L. borgpetersenii* | Hardjo-bovis | JB197 | 10^4^ | <10^8^ |
|  | Hardjo-bovis | 203^*^ | ND | >10^8^ |

^£^ Results are the average of two independent experiments

^*^ This strain was described to cause only kidney colonization in hamsters following intraperitoneal (IP) infection ^17^, for that reason there was no LD_50_ for IP route. However, in two experiments the fatality rate was 25% after infection with 10^8^ leptospires by conjunctival (CJ) infection
