# Supplementary material for: A live attenuated-vaccine model confers cross-protective immunity against different species of the Leptospira genus
Source: eLife. 2021 Jan 26;10:e64166. doi: 10.7554/eLife.64166 (PMC7837694; doi:10.7554/eLife.64166)
Supplement: Supplementary file 2. [file elife-64166-supp2.docx]

**Supplementary Table 2.** Efficacy of the immunization with a dose of 10^7^ leptospires of the attenuated L1-130 *fcpA*^-^ mutant in hamsters followed by challenge with 10^8^ leptospires with homologous or heterologous strains by conjunctival route

| **Vaccine^£^** | **Challenge** | | **Expt.^*^** | **Median days**  **for death (range)** | | **% Vaccine protection** | | | | **% Vaccine Efficacy overall**  **(95% CI)^¶^** | |
| --- | --- | --- | --- | --- | --- | --- | --- | --- | --- | --- | --- |
|  |  |  |  |  |  | **Death**  **(No. survivors/total)** | | **Colonization**  **(No. negative/total)** | |  |  |
|  | **Species** | **Serovar/Strain** |  | **Vaccine** | **PBS**  **control** | **Vaccine** | **PBS control** | **Vaccine** | **PBS control** | **Death** | **Colonization** |
| *fcpA^-^* | *L. interrogans* | Copenhageni Fiocruz L1-130 | 1 | - | 9 (8-11) | 100 (9/9) | - (0/9) | 77.8 (7/9) | - (0/9) | 100  (89–100) | 80.6  (63.7-90.8) |
|  |  |  | 2 | - | 8 (7-9) | 100 (9/9) | - (0/8) | 55.5 (5/9) | - (0/8) |  |  |
|  |  |  | 3 | - | 10 (8-11) | 100 (7/7) | - (0/7) | 100 (7/7) | - (0/7) |  |  |
|  |  |  | 4 | - | 8 (8-9) | 100 (6/6) | - (0/6) | 100 (6/6) | - (0/6) |  |  |
|  |  | Manilae L495 | 1 | - | 8 (7-10) | 100 (9/9) | - (0/9) | 33.3 (3/9) | - (0/9) | 100  (86.7–100) | 20  (8.9-39.1) |
|  |  |  | 2 | - | 8 (8-9) | 100 (9/9) | - (0/8) | 11 (1/9) | - (0/8) |  |  |
|  |  |  | 4 | - | 9 (9-12) | 100 (7/7) | - (0/7) | 14.3 (1/7) | - (0/7) |  |  |
|  |  | Pomona  PO-06-047 | 2 | - | 10 (9-18) | 100 (8/8) | - (0/8) | 0 (0/8) | - (0/8) | 100  (80.6-100) | 0  (0–19.4) |
|  |  |  | 3 | - | 10.5 (9-13) | 100 (8/8) | - (0/8) | 0 (0/8) | - (0/8) |  |  |
|  |  | Canicola Kito | 3 | - | 10.5 (10-15) | 100 (8/8) | - (0/8) | 37.5 (3/8) | - (0/8) | 100  (79.6-100) | 26.7  (10.9-52) |
|  |  |  | 4 | - | 11 (9-11) | 100 (7/7) | - (0/7) | 14.3 (1/7) | - (0/7) |  |  |
|  | *L. kirschneri* | Grippotyphosa RM-52 | 3 | - | 9 (8-11) | 100 (7/7) | - (0/7) | 28.6 (2/7) | - (0/7) | 100  (78.5-100) | 34  (16.3-61.2) |
|  |  |  | 4 | - | 9 (8-11) | 100 (7/7) | - (0/7) | 42.9 (3/7) | - (0/7) |  |  |
|  | *L. borgpetersenii* | Hardjo-bovis JB197 | 3 | - | 7 (6-10) | 100 (7/7) | - (0/7) | 0 (0/7) | - (0/7) | 100  (78.5 – 100) | 0  (0-21.5) |
|  |  |  | 4 | - | 11 (9-12) | 100 (7/7) | - (0/7) | 0 (0/7) | - (0/7) |  |  |
|  |  | Hardjo-bovis 203 | 3 | - | 12 (12) | 100 (7/7) | - (5/7)^§^ | 71.4 (5/7) | - (0/7) | 100  (43.9-100)^§^ | 35.7  (16.3-61.2) |
|  |  |  | 4 | - | 14 (14) | 100 (7/7) | - (6/7)^§^ | 0 (0/7) | - (0/7) |  |  |
| Heat-killed | *L. interrogans* | Copenhageni Fiocruz L1-130 | 1 | 8 (0.58) | 9 (8-9) | 66.7 (6/9) | - (0/9) | 55.5 (5/9) | - (0/9) | 58.9  (36-78.4) | 35.3  (17.3-58.7) |
|  |  |  | 2 | 8.5 (3.37) | 8 (8-15) | 50 (4/8) | - (0/8) | 12.5 (1/8) | - (0/8) |  |  |
|  |  | Manilae L495 | 1 | 9 (0.7) | 8 (8-10) | 11 (1/9) | - (0/9) | 0 (0/9) | - (0/9) | 11.8  (3.3-34.3) | 0  (0–18.4) |
|  |  |  | 2 | 8 (1.03) | 8 (7-9) | 12.5 (1/8) | - (0/8) | 0 (0/8) | - (0/8) |  |  |

^£^ *Leptospira interrogans* serovar Copenhageni strain Fiocruz L1-130

^*^ The experiment identification refers to the group of strains that were tested at the same time

^¶^ Calculations based on frequency of outcomes compared to PBS-immunized animals

^§^ Only 3/14 animals were euthanized due to clinical signs within the control group
