# Supplementary material for: A live attenuated-vaccine model confers cross-protective immunity against different species of the Leptospira genus
Source: eLife. 2021 Jan 26;10:e64166. doi: 10.7554/eLife.64166 (PMC7837694; doi:10.7554/eLife.64166)
Supplement: Supplementary file 3. [file elife-64166-supp3.docx]

**Supplementary Table 3.** Efficacy of the immunization with different doses of the attenuated L1-130 *fcpA*^-^ mutant in mice followed by challenge with 10^8^ leptospires of heterologous strain by intraperitoneal route

| **Vaccine^£^** | **Challenge** | | **Vaccine Dose^£^** | **Expt.^*^** | **Median days for death (range)** | | **% Vaccine protection** | | | | **% Vaccine Efficacy overall (95% CI)^¶^** | |
| --- | --- | --- | --- | --- | --- | --- | --- | --- | --- | --- | --- | --- |
|  |  |  |  |  |  |  | **Death (No. survivors/total)** | | **Colonization  (No. negative/total)** | |  |  |
|  | **Species** | **Serovar/Strain** |  |  | **Vaccine** | **PBS**  **control** | **Vaccine** | **PBS**  **control** | **Vaccine** | **PBS**  **control** | **Death** | **Colonization** |
| *fcpA^-^* | *L. interrogans* | Manilae L495 | 10^7^ | 1 | - | 3 (3-6) | 100 (8/8) | - (0/8) | 100 (8/8) | - (0/8) | 100  (82.4–100) | 100  (82.4-100) |
|  |  |  |  | 2 | - | 3 (3) | 100 (6/6) | - (0/6) | 100 (6/6) | - (0/6) |  |  |
|  |  |  |  | 3 | - | 3 (3-4) | 100 (4/4) | - (0/4) | 100 (4/4) | - (0/4) |  |  |
|  |  |  | 10^5^ | 2 | - | 3 (3) | 100 (4/4) | - (0/4) | 50 (2/4) | - (0/4) | 100  (67.6–100) | 50  (21.5-78.5) |
|  |  |  |  | 3 | - | 3 (3-4) | 100 (4/4) | - (0/4) | 50 (2/4) | - (0/4) |  |  |
|  |  |  | 10^3^ | 2 | - | 3 (3) | 100 (4/4) | - (0/4) | 25 (1/4) | - (0/4) | 100  (67.6-100) | 25  (7.1–59.1) |
|  |  |  |  | 3 | - | 3 (3-4) | 100 (4/4) | - (0/4) | 25 (1/4) | - (0/4) |  |  |
|  |  |  | 10^2^ | 2 | 3.5 (3-4) | 3 (3) | 50 (2/4) | - (0/4) | 0 (0/4) | - (0/4) | 50  (21.5-78.5) | 0  (0-32.4) |
|  |  |  |  | 3 | 3.5 (3-4) | 3 (3-4) | 50 (2/4) | - (0/4) | 0 (0/4) | - (0/4) |  |  |
|  |  |  | 10^1^ | 2 | 3.5 (3-4) | 3 (3) | 0 (0/4) | - (0/4) | 0 (0/4) | - (0/4) | 0  (0-32.4) | 0  (0-32.4) |
|  |  |  |  | 3 | 4 (3-5) | 3 (3-4) | 0 (0/4) | - (0/4) | 0 (0/4) | - (0/4) |  |  |

^£^ *Leptospira interrogans* serovar Copenhageni strain Fiocruz L1-130

^*^ The experiment identification refers to the group of strains that were tested at the same time

**^¶^** Calculations based on frequency of outcomes compared to PBS-immunized animals
